# Urinary Concentrations of Four Parabens in the U.S. Population: NHANES 2005–2006

**DOI:** 10.1289/ehp.0901560

**Published:** 2010-01-04

**Authors:** Antonia M. Calafat, Xiaoyun Ye, Lee-Yang Wong, Amber M. Bishop, Larry L. Needham

**Affiliations:** Division of Laboratory Sciences, National Center for Environmental Health, Centers for Disease Control and Prevention, Atlanta, Georgia, USA

**Keywords:** biomonitoring, exposure, human, NHANES, *p*-hydroxybenzoic acid esters, urine

## Abstract

**Background:**

Parabens are widely used as antimicrobial preservatives in cosmetics, pharmaceuticals, and food and beverage processing.

**Objectives:**

We assessed exposure to methyl, ethyl, propyl, and butyl parabens in a representative sample of persons ≥ 6 years of age in the U.S. general population from the 2005–2006 National Health and Nutrition Examination Survey.

**Methods:**

We analyzed 2,548 urine samples by using online solid-phase extraction coupled to isotope dilution–high-performance liquid chromatography/tandem mass spectrometry.

**Results:**

We detected methyl paraben (MP) and propyl paraben (PP) in 99.1% and 92.7% of the samples, respectively. We detected ethyl (42.4%) and butyl (47%) parabens less frequently and at median concentrations at least one order of magnitude lower than MP (63.5 μg/L) and PP (8.7 μg/L). Least-square geometric mean (LSGM) concentrations of MP were significantly higher (*p* ≤ 0.01) among non-Hispanic blacks than among non-Hispanic whites except at older ages (≥ 60 years). Adolescent and adult females had significantly higher (*p* < 0.01) LSGM concentrations of MP and PP than did adolescent and adult males. Females were more likely than males [adjusted odds ratios (ORs) and 95% confidence intervals (CIs): MP, 3.2 (2.99–5.27); PP, 4.19 (2.34–7.49)] and non-Hispanic blacks were more likely than non-Hispanic whites [MP, 4.99 (2.62–9.50); PP, 3.6 (1.86–7.05)] to have concentrations above the 95th percentile.

**Conclusions:**

The general U.S. population was exposed to several parabens during 2005–2006. Differences in the urinary concentrations of MP and PP by sex and race/ethnicity likely reflect the use of personal care products containing these compounds.

Parabens are esters of *p*-hydroxybenzoic acid. They are used as antimicrobial preservatives, especially against molds and yeast, in cosmetics, pharmaceuticals, and food and beverage processing (Andersen 2008; Elder 1984). Parabens are popular preservatives because of their low toxicity and cost, their broad inertness, and their worldwide regulatory acceptance (Soni et al. 2005). Methyl paraben (MP) and propyl paraben (PP) are two of the most used parabens (Andersen 2008).

Parabens are not mutagenic (Elder 1984), but their potential estrogenic activity—which is orders of magnitude lower than that of estrogen (Andersen 2008; Golden et al. 2005)—has raised some concerns. The presence of parabens in human breast tumors (Darbre et al. 2004) triggered a debate about their use in cosmetics, particularly in underarm deodorants and antiperspirants, and the incidence of breast cancer (Darbre 2006; Darbre and Harvey 2008; Golden et al. 2005; Harvey and Darbre 2004). However, the Scientific Committee on Consumer Products (SCCP) of the European Commission concluded that there was no evidence that using paraben-containing underarm cosmetics increased the demonstrable risk of breast cancer (SCCP 2005). In 2003, the Cosmetic Ingredient Review (CIR) expert panel, part of a program established by the Cosmetics, Toiletry, and Fragrance Association, with support from the U.S. Food and Drug Administration and the Consumer Federation of America, decided to reevaluate the safety of parabens (Bergfeld et al. 2005) and to assess their link to endocrine disruption. In 2006, the CIR panel concluded that methyl, ethyl, propyl, isopropyl, butyl, isobutyl, and benzyl parabens are safe as cosmetic ingredients in the practices of use and use concentrations (up to 0.4% if used alone, up to 0.8% in mixtures) (Andersen 2008). Nonetheless, the National Institute of Environmental Health Sciences has recommended butyl paraben (BP) for toxicologic evaluation because of its widespread use, its potential reproductive toxicity, and the lack of adequate toxicologic data (National Toxicology Program 2004, 2006). Furthermore, SCCP recently concluded that the safety assessment of PP and BP could not yet be finalized on the basis of available data (SCCP 2008).

Exposure to parabens may occur through inhalation, dermal contact, and ingestion (El Hussein et al. 2007; Soni et al. 2005), and metabolism may differ, depending upon the exposure route (Bando et al. 1997; Soni et al. 2005). Also, metabolism and uptake from human skin may be lower than in other species (Harville et al. 2007; Jewell et al. 2007a; Prusakiewicz et al. 2006), and interindividual differences among humans in dermal metabolic capacity exist (Jewell et al. 2007b). Parabens can be hydrolyzed to *p*-hydroxybenzoic acid, which can be conjugated before urinary excretion (Kiwada et al. 1979, 1980), but they can also be excreted as intact esters (Ye et al. 2006a). The fraction of the parabens excreted in the urine as the parent paraben (free or conjugated) versus the fraction excreted as *p*-hydroxybenzoic acid is unknown. In humans, *p*-hydroxybenzoic acid, a nonspecific metabolite of all parabens, and its conjugates in urine are not optimal biomarkers of exposure to parabens, known to have quite different bioactivities. By contrast, the concentrations of total (free plus conjugated) urinary species of the parent parabens can be used as valid human exposure biomarkers (Ye et al. 2006a).

Our evaluation of the urinary concentrations of parabens among 100 adults with no known occupational exposure to the compounds showed widespread exposure to these chemicals (Ye et al. 2006a). Here we report the first nationally representative data on the total concentrations of four parabens in urine among those in the U.S. general population ≥ 6 years of age, stratified by age group, sex, and race/ethnicity.

## Methods

Since 1999, the Centers for Disease Control and Prevention (CDC) has conducted annually the National Health and Nutrition Examination Survey (NHANES) to measure the health and nutritional status of the civilian noninstitutionalized U.S. population ≥ 2 months of age (CDC 2009b). NHANES includes household interviews, standardized physical examinations, and collection of medical histories and biologic specimens. Some of these specimens are used to assess exposure to environmental chemicals (CDC 2009b). For this study, 2,548 urine specimens were collected from a one-third subset of participants ≥ 6 years of age. The representative design of the survey was maintained because the subset was random. The National Centers for Health Statistics Institutional Review Board reviewed and approved the study protocol. All participants gave informed written consent; parents or guardians provided consent for participants < 18 years of age.

Participants provided one spot urine sample during one of three daily examination sessions. The samples were shipped on dry ice to CDC’s National Center for Environmental Health and stored at temperatures below −20°C until analyzed. We measured the concentrations of total parabens, described in detail elsewhere (Ye et al. 2006b). We first hydrolyzed the conjugated species of parabens in 100 μL urine by use of β-glucuronidase/sulfatase (*Helix pomatia*, H1; Sigma Aldrich Laboratories, Inc., St. Louis, MO). The parabens were preconcentrated by online solid-phase extraction, separated from other urine components by reversed-phase high-performance liquid chromatography, and detected by atmospheric pressure chemical ionization–isotope dilution/tandem mass spectrometry with peak focusing (Ye et al. 2006b). The limits of detection (LODs) in synthetic urine, calculated as 3S_0_, where S_0_ is the standard deviation as the concentration approaches zero (Taylor 1987), were 1.0 μg/L [MP, ethyl paraben (EP)] and 0.2 μg/L (PP, BP). We prepared low-concentration (2.2–9.0 μg/L) and high-concentration (10.5–53.8 μg/L) quality control materials (QCLs and QCHs, respectively) with pooled human urine that was analyzed with standards, reagent blanks, and NHANES samples. The precision of measurements, expressed as the relative standard deviation of 55–66 measures, depending on the analyte, was 5.8–12.1% for QCLs and 4.4–5.6% for QCHs.

We used SAS (version 9.1.3; SAS Institute Inc., Cary, NC) and SUDAAN (version 10; Research Triangle Institute, Research Triangle Park, NC) to perform statistical analyses. SUDAAN calculates variance estimates after incorporating the sample population weights, used to produce estimates that are representative of the U.S. population, which account for nonresponse rates, unequal selection probabilities, and planned oversampling of certain subgroups resulting from the complex multistage probability design of NHANES. We stratified age, reported in years at the last birthday, in four groups (6–11, 12–19, 20–59, and ≥ 60 years). On the basis of self-reported data, we categorized race/ethnicity as non-Hispanic black, non-Hispanic white, and Mexican American. Participants not defined by these racial/ethnic categories were included only in the total population estimate. For each age, sex, and race/ethnic group, we calculated the geometric means (if the overall weighted frequency of detection was > 60%) and distribution percentiles for both volume-based (micrograms per liter) and creatinine-corrected (micrograms per gram creatinine) concentrations. For concentrations below the LOD, we used a value equal to the LOD divided by the square root of 2 in univariate and multivariate analyses, as recommended for examining NHANES data (CDC 2009a).

We used analysis of covariance to examine whether several variables, selected on the basis of statistical, demographic, and biological considerations, were associated with the log-transformed urine concentrations of MP and PP. We considered age (continuous), sex, race/ethnicity, creatinine concentration, household income (described below), and examination session (i.e., morning, afternoon, evening). On the basis of questionnaire responses, annual household income was available in increments of $5,000 (ranging from < $5,000 to > $75,000). We categorized income as < $20,000, $20,000–$45,000, and > $45,000 to obtain a comparable number of participants per group. For the multiple regression models, we used the variables described previously and all their possible two-way interactions to calculate the adjusted least-square geometric mean (LSGM) concentrations (micrograms per liter) of MP and PP. These variables were log transformed because the distributions of concentrations of these parabens and creatinine were skewed.

To arrive at the final model, we used backward elimination with SUDAAN to remove the nonsignificant interactions one at a time for each analyte. Nonsignificant main effects were then removed one at a time, and the model was rerun to determine whether the β-coefficients for significant main effects or interactions changed by > 10%. If any did, we retained the nonsignificant main effect in the model. Once the backward procedure was completed, main effects and interactions were added back into the model one at a time to determine whether any were significant (*p* < 0.05). We retained those that were in the final model.

We also conducted weighted univariate and multiple logistic regressions to examine the association of MP and PP concentrations above the 95th percentile with sex, age group, race/ethnicity, household income, and examination session.

## Results

We detected MP at concentrations ranging from 1.0 to 17,300 μg/L in 99.1% of persons examined. We detected PP in 92.7% of persons at concentrations of 0.2–7,210 μg/L. We detected EP and BP less frequently and at lower concentration ranges (EP: 42.4%, 1.0–1,110 μg/L; BP: 47%, 0.2–1,240 μg/L) than MP and PP. The geometric mean and 95th percentile concentrations were 56.4 μg/L (55.0 μg/g creatinine) and 974 μg/L (902 μg/g creatinine) for MP, and 7.72 μg/L (7.53 μg/g creatinine) and 299 μg/L (263 μg/g creatinine) for PP, respectively (Tables 1 and 2). Because the weighted frequency of detection was < 60% for BP (except for females) and EP, we did not calculate the geometric mean (Tables 3 and 4) or conduct multivariate analysis.

We found that the concentrations of MP and PP were highly correlated [Pearson correlation coefficient (*R*) = 0.62350, *p* < 0.0001]. We also observed significant (*p* < 0.0001), but not as strong (*R* = 0.49637), correlations between the concentrations of BP and EP. The correlations between MP or PP and both BP and EP were weaker (*R* = 0.17911–0.34222) yet statistically significant (*p* < 0.0001).

**Figure 1:**
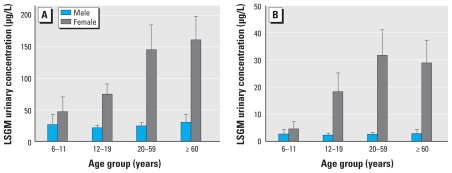
LSGM urinary concentrations by age and sex: (*A*) MP; (*B*) PP. Error bars indicate 95% CIs.

**Figure 2:**
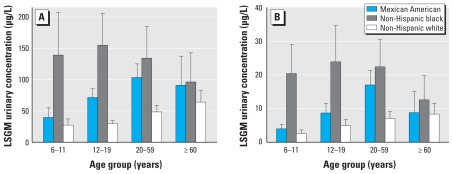
LSGM urinary concentrations by age and race/ethnicity: (*A*) MP; (*B*) PP. Error bars indicate 95% CIs.

The final MP and PP models included log creatinine, income, and the interaction terms sex × race/ethnicity (only for PP), sex × age (*p* < 0.01), and age × race/ethnicity (*p* < 0.01) (Table 5). For both parabens, LSGM concentrations for persons in the high household income category were significantly higher than for those in the low (*p* < 0.01) and medium (MP, *p* = 0.05; PP, *p* < 0.01) categories (Table 5). Females had significantly higher concentrations (*p* < 0.01) of both parabens than did males for all age groups, except children (MP, *p* = 0.11; PP, *p* = 0.81) (Figure 1); females also had significantly higher PP concentrations (*p* < 0.01) than did males, regardless of race/ethnicity (Table 5). Regardless of sex, adults ≥ 60 years of age had significantly higher LSGM concentrations of both parabens than did children (MP, *p* = 0.05; PP, *p* < 0.01) and adolescents (*p* = 0.03). Children had significantly lower LSGM concentrations than did adults 20–59 years of age (MP, *p* = 0.03; PP, *p* < 0.01) and adolescents (*p* = 0.04 for PP only) (Table 5, Figure 1). Except for persons ≥ 60 years of age, LSGM concentrations of MP were significantly higher for non-Hispanic blacks than for non-Hispanic whites (*p* < 0.01; Table 5, Figure 2). Similarly, non-Hispanic black children and adolescents (*p* < 0.01) had significantly higher concentrations of MP than Mexican Americans. We found the same pattern for PP (Table 5), except that the differences for adolescents were only of borderline significance (*p* = 0.07). Mexican Americans had higher LSGM concentrations of both MP and PP than did non-Hispanic whites (Table 5), but the differences reached statistical significance (*p* < 0.01) only among adults and adolescents for MP. Among Mexican Americans, adults had significantly higher LSGM concentrations (*p* < 0.01) of both MP and PP than did children and adolescents; the LSGM concentrations among children were significantly lower than those for adults ≥ 60 years of age (*p* < 0.01) and adolescents (MP, *p* = 0.01; PP, *p* < 0.01). Among non-Hispanic whites, adults and older adults had significantly higher LSGM concentrations than did adolescents (MP, *p* < 0.01; PP, *p* = 0.03 for adults and *p* < 0.01 for older adults) and children (*p* < 0.01) (Table 5). Among non-Hispanic blacks, adults had significantly higher LSGM concentrations of MP and PP than did children (MP, *p* = 0.02; PP, *p* < 0.01), and adolescents also had significantly higher LSGM concentrations of PP than did children (*p* = 0.01) (Table 5).

For both MP and PP, the likelihood of having a urinary concentration above the 95th percentile (an arbitrary value selected as an example of higher than average concentrations) was significantly associated with sex (*p* < 0.01) and race (*p* < 0.01) but not with age, income, or examination session. Females were about three to four times more likely than males to have urinary concentrations of MP and PP above the 95th percentile [adjusted odds ratios (ORs) with 95% confidence intervals (CIs): MP, 3.2 (2.99–5.27); PP, 4.19 (2.34–7.49)]. Compared with non-Hispanic whites, the likelihood of having urinary concentrations of MP or PP at or above the 95th percentile was two to five times higher for non-Hispanic blacks [MP, 4.99 (2.62–9.50); PP, 3.6 (1.86–7.05)] and for Mexican Americans (MP, 2.03 (1.30–3.17); PP, 2.56 (1.56–4.24)]. Non-Hispanic blacks were about 2.5 times more likely than Mexican Americans to have MP concentrations above the 95th percentile [2.45 (1.41–4.28)], but we found no significant difference (*p* = 0.13) between non-Hispanic blacks and Mexican Americans for PP.

## Discussion

We detected concentrations of free plus conjugated species of MP and PP in urine in > 92% of the samples examined; we detected EP and BP in about 50%. The high frequency of detection of MP and PP most likely resulted from their wide use in food products (Soni et al. 2005) and in common personal care products (e.g., lotions, cosmetics, hair preparations) (Andersen 2008). The range of urinary concentrations spanning up to three orders of magnitude (Tables 1–4) may be related to lifestyle factors, including diet, that result in exposure differences and/or to individual variations in bioavailability, distribution kinetics, or metabolism of the parabens. These factors are important in interpreting biomonitoring data for other chemicals found in personal care products, such as the bactericide triclosan (Calafat et al. 2008b; Sandborgh-Englund et al. 2006) and the sunscreen agent benzophenone-3 (Calafat et al. 2008a). All these factors as well as the timing of sample collection will affect the urinary concentrations of parabens on an individual basis. On a population basis (e.g., NHANES), however, the wide range of concentrations observed would represent an average exposure scenario (i.e., a paraben urinary concentration in the upper percentiles resulting from the collection of the urine soon after a person’s paraben-related activity may be offset by a concentration in the lower percentiles of another person who provided a urine sample shortly before conducting the same activity).

We observed moderate correlations between the concentrations of EP and BP, and much weaker correlations between either of these parabens and MP or PP. These findings suggest a potential common source(s) of exposure for EP and BP, which was not the case for MP and PP. The concentrations of MP and PP were highly correlated, most likely because they are the two most common parabens (Soni et al. 2005) and may be used in combination in many commercial applications, including food, pharmaceuticals, and personal care products.

We previously reported the urinary concentrations of free plus conjugated species of several parabens in a convenience sample of 100 adults during 2003–2005 (Ye et al. 2006a). The frequencies of detection were comparable among the sample (MP, 99%; PP, 96%; EP, 58%; BP, 69%) and NHANES 2005–2006 (MP, 99.1%; PP, 92.7%; EP, 42%; BP, 47%). In addition, the median concentrations were practically the same for PP, slightly higher for MP, and lower for BP and EP in the NHANES 2005–2006 sample compared with the convenience population. Although exposure to these parabens (assessed by the urinary concentrations) may have changed, these two data sets are not directly comparable for establishing temporal exposure trends because of the small sample size and lack of national representativeness of the convenience sample. Nonetheless, both data sets confirm considerable human exposure to parabens and could potentially be used to derive internal dose exposure estimates. Of interest, the convenience biomonitoring samples have already been used to estimate internal dose of parabens (Cowan-Ellsberry and Robison 2009). Unfortunately, data are lacking regarding human metabolism of parabens, particularly of the fraction of the paraben excreted in the urine as the parent paraben (free or conjugated) versus the fraction excreted as *p*-hydroxybenzoic acid. This information, including a better understanding of the possible differences in metabolism by exposure route in humans, is needed to adequately link paraben urinary biomarker measurements to exposure and to internal dose.

We report here for the first time the concentrations of parabens among children and adolescents. Our data confirm that exposure occurs at these younger ages. Further, the apparent lower exposure among the younger segments of this NHANES 2005–2006 population (reflected in lower urinary concentrations than for adults) is likely associated with lifestyle. For example, in general, adults are more likely to use pharmaceuticals and personal care products than are children. We observed a similar age pattern for monoethyl phthalate (Silva et al. 2004), a metabolite of diethyl phthalate (DEP) for which a primary source of exposure may be the routine use of personal care products (Duty et al. 2005).

Although parabens are nonpersistent chemicals that are excreted from the body within hours after exposure (Janjua et al. 2008), examination session used as a surrogate for the time of urine collection was not a significant factor in explaining the variance of the urinary concentrations of the parabens. In contrast, we observed important differences in concentrations on the basis of demographic characteristics. Specifically, the LSGM MP and PP concentrations were significantly greater among people in the high household income category than among those in the medium- and low-income categories (Table 5), suggesting that the use of pharmaceuticals and personal care products according to socioeconomic status may affect paraben exposure.

Of interest, we observed that LSGM concentrations of MP and PP were highly dependent upon sex, age, and race/ethnicity (Table 5, Figures 1 and 2), as we have reported for other compounds (or their metabolites) found in personal care products, such as DEP (Silva et al. 2004) and benzophenone-3 (Calafat et al. 2008a). The higher concentrations of MP and PP found among women than among men were likely attributable to women’s increased use of personal care products, such as cosmetics and lotions. Non-Hispanic black children and adolescents had LSGM concentrations of MP and PP that were higher than or very similar to the concentrations in non-Hispanic black adults; non-Hispanic blacks had much higher MP and PP concentrations than did the other two race/ethnicity groups, particularly among children, adolescents, and adults 20–59 years old. These differences may result from increased, continuous, or prolonged use of beauty, hair, and/or skin care products specifically marketed to this population in whom the use often begins at a young age. The less dramatic differences by race/ethnicity among older adults may be explained by increased use of pharmaceuticals regardless of race/ethnicity that may compensate for differences in personal care products use. Because MP and PP are also used in food products, we cannot rule out that potential differences in diet, should they exist, may have also contributed to the differences in urinary concentrations of MP and PP among the various demographic groups examined.

Identifying populations in the highest exposure category (i.e., with concentrations above the 95th percentile) is an important consideration for public health. Our data suggest that females, non-Hispanic blacks, and, to a lesser extent, Mexican Americans have higher exposures to MP and PP than do other demographic segments of the general population. Specifically, females and non-Hispanic blacks were more likely to exhibit concentrations of MP and PP above the 95th percentile than were males, non-Hispanic whites, or Mexican Americans. In particular, females were 3.2 times more likely than males, and non-Hispanic blacks were about 5 times more likely than non-Hispanic whites and 2.5 times more likely than Mexican Americans, to have MP concentrations above the 95th percentile. Mexican Americans were about twice as likely as non-Hispanic whites to present MP concentrations above the 95th percentile. Similarly, for PP, females were 4.2 times more likely than males, and non-Hispanic whites were 3.6 times less likely than non-Hispanic blacks and 2.56 times less likely than Mexican Americans, to have MP concentrations above the 95th percentile. The likelihood of presenting PP concentrations above the 95th percentile did not differ (*p* = 0.13) between Mexican Americans and non-Hispanic blacks. Age was not significantly associated with having concentrations above the 95th percentile for either MP or PP.

In summary, we found significant differences in concentrations of parabens across demographic groups, particularly those associated with sex and race/ethnicity. These data can be used to establish a nationally representative baseline assessment of exposure—a baseline against which the concentrations of these parabens in future populations can be compared in order to identify exposure trends. These NHANES 2005–2006 data may also be useful in a risk assessment of parabens if warranted by toxicologic or epidemiologic studies.

## Figures and Tables

**Table 1:** Geometric means and selected percentiles (95% CIs) of MP concentrations in urine for the U.S. population ≥ 6 years of age: NHANES 2005–2006.

Variable	Geometric mean	Percentile						Sample size
		10th	25th	50th	75th	90th	95th	
All								
μg/L	56.4 (46.9–67.9)	5.60 (4.70–6.90)	13.4 (10.6–17.6)	63.5 (47.9–74.5)	216 (168–282)	560 (432–777)	974 (827–1,110)	2,548
μg/g creatinine	55.0 (46.8–64.6)	5.38 (4.55–6.10)	12.9 (11.0–15.9)	58.8 (47.0–73.4)	221 (170–264)	527 (426–685)	902 (753–995)	2,548

Age group (years)								
6–11								
μg/L	33.5 (25.6–43.8)	5.20 (4.60–5.60)	9.60 (7.80–13.8)	25.0 (19.4–35.3)	86.7 (61.5–153)	289 (238–868)	1,560 (389–2,090)	356
μg/g creatinine	36.8 (27.9–48.7)	5.79 (3.97–7.80)	10.5 (7.62–17.4)	26.9 (21.0–36.6)	108 (55.9–164)	259 (195–753)	1,540 (313–2,510)	356
12–19								
μg/L	53.8 (43.9–66.0)	6.40 (5.20–7.20)	14.2 (11.9–18.7)	53.5 (39.4–65.7)	192 (163–264)	491 (380–720)	901 (571–1,210)	702
μg/g creatinine	40.1 (33.7–47.8)	4.54 (3.75–5.43)	10.5 (8.17–12.5)	41.7 (31.5–56.3)	160 (129–178)	362 (280–428)	549 (434–656)	702
20–59								
μg/L	61.0 (47.8–78.0)	5.70 (4.10–7.30)	15.3 (10.7–21.1)	71.3 (57.3–92.1)	221 (168–322)	557 (412–814)	949 (742–1,120)	1,040
μg/g creatinine	58.6 (47.9–71.7)	5.39 (4.03–6.84)	14.2 (11.8–18.4)	65.9 (48.5–89.7)	242 (170–295)	561 (416–733)	910 (706–1,010)	1,040
≥60								
μg/L	58.0 (49.3–68.2)	6.10 (4.70–7.70)	11.6 (8.90–14.7)	59.8 (42.0–90.0)	243 (177–347)	661 (490–877)	1,040 (877–1,200)	450
μg/g creatinine	67.5 (55.1–82.7)	5.64 (4.61–7.27)	14.2 (10.3–18.5)	86.4 (58.6–109)	326 (221–397)	709 (508–824)	988 (799–1,550)	450

Sex								
Female								
μg/L	104 (80.8–135)	9.10 (6.20–14.1)	35.4 (22.1–48.3)	137 (93.0–168)	356 (279–427)	842 (660–974)	1,110 (956–1,335)	1,278
μg/g creatinine	123 (99.3–152)	12.9 (9.03–17.7)	43.2 (29.1–61.6)	147 (111–196)	377 (319–445)	788 (676–910)	1,050 (937–1,290)	1,278
Male								
μg/L	29.8 (24.8–35.8)	4.10 (2.90–5.60)	8.30 (7.20–10.1)	23.7 (19.4–29.4)	97.6 (78.1–121)	299 (229–355)	491 (385–743)	1,270
μg/g creatinine	23.9 (20.6–27.8)	3.73 (2.95–4.57)	7.48 (6.16–8.40)	21.1 (17.4–25.3)	72.1 (58.0–89.5)	209 (160–260)	368 (262–498)	1,270

Race/ethnicity^a^								
Mexican American								
μg/L	78.2 (61.0–100)	8.40 (5.30–11.3)	21.8 (16.1–30.1)	86.3 (64.7–121)	301 (233–349)	742 (474–896)	1,100 (896–1,170)	637
μg/g creatinine	70.3 (57.1–86.7)	7.31 (4.88–10.2)	21.0 (13.6–27.7)	85.8 (67.2–106)	249 (191–303)	515 (431–656)	870 (656–1,080)	637
Non-Hispanic black								
μg/L	174 (133–229)	16.0 (12.7–20.4)	54.2 (31.4–91.6)	216 (150–306)	616 (465–789)	1,180 (1,010–1,480)	1,690 (1,210–2,880)	678
μg/g creatinine	122 (93.0–161)	12.2 (8.30–17.0)	42.4 (24.5–71.1)	161 (98.2–225)	377 (334–462)	792 (581–1,100)	1,220 (916–1,590)	678
Non-Hispanic white								
μg/L	43.8 (36.7–52.3)	5.00 (3.70–6.20)	10.7 (8.60–13.6)	44.3 (36.7–58.2)	165 (132–209)	412 (320–520)	806 (485–974)	1,038
μg/g creatinine	46.1 (39.0–54.4)	5.11 (4.16–5.57)	10.9 (9.29–13.0)	45.6 (34.5–59.7)	170 (142–234)	447 (339–706)	814 (640–995)	1,038

CI, confidence interval.

a Participants not defined by the three racial/ethnic groups shown were included only in the total population estimate. LOD = 1.0 μg/L.

**Table 2:** Geometric means and selected percentiles (95% CIs) of PP concentrations in urine for the U.S. population ≥ 6 years of age: NHANES 2005–2006.

Variable	Geometric mean	Percentile						Sample size
		10th	25th	50th	75th	90th	95th	
All								
μg/L	7.91 (6.41–9.77)	0.300 (0.200–0.400)	1.20 (0.900–1.60)	8.70 (5.90–12.4)	48.9 (36.6–64.8)	160 (129–200)	299 (237–354)	2,548
μg/g creatinine	7.71 (6.40–9.30)	0.344 (0.278–0.422)	1.17 (0.896–1.58)	8.27 (5.87–11.3)	52.9 (41.6–66.1)	161 (140–178)	263 (226–288)	2,548

Age group (years)								
6–11								
μg/L	3.41 (2.43–4.77)	0.300 (< LOD–0.500)	0.800 (0.700–1.20)	2.50 (1.60–4.30)	11.1 (7.10–21.0)	47.5 (30.0–91.9)	125 (55.8–383)	356
μg/g creatinine	3.75 (2.67–5.26)	0.412 (< LOD–0.684)	1.01 (0.682–1.51)	2.70 (1.89–4.43)	11.5 (8.07–16.0)	52.1 (22.9–100)	121 (55.5–352)	356
12–19								
μg/L	8.16 (5.70–11.7)	0.400 (< LOD–0.500)	1.50 (0.900–2.60)	8.40 (5.00–13.9)	46.4 (24.1–68.5)	165 (96.7–222)	310 (201–361)	702
μg/g creatinine	6.08 (4.36–8.49)	0.350 (0.223–0.571)	1.23 (0.676–1.82)	5.44 (3.03–10.5)	33.0 (20.7–48.7)	116 (81.9–161)	175 (140–239)	702
20–59								
μg/L	8.99 (6.88–11.7)	0.400 (< LOD–0.600)	1.50 (1.10–2.00)	11.3 (7.50–15.3)	52.2 (38.2–75.7)	162 (118–240)	317 (215–370)	1,040
μg/g creatinine	8.63 (6.84–10.9)	0.350 (< LOD–0.473)	1.33 (0.972–1.81)	9.79 (6.57–15.2)	58.1 (42.9–80.4)	178 (144–198)	265 (226–306)	1,040
≥ 60								
μg/L	7.67 (6.00–9.80)	0.200 (< LOD–0.400)	0.900 (0.600–1.40)	9.70 (5.70–13.7)	64.5 (39.4–87.6)	190 (134–251)	295 (219–386)	450
μg/g creatinine	8.92 (6.93–11.5)	0.268 (< LOD–0.378)	0.891 (0.571–1.28)	12.9 (8.82–22.7)	77.6 (60.0–106)	177 (142–228)	278 (199–309)	450

Sex								
Female								
μg/L	20.4 (16.0–25.9)	0.900 (0.700–1.40)	5.30 (3.20–8.30)	29.1 (21.6–37.5)	93.0 (75.7–129)	254 (193–318)	357 (318–395)	1,278
μg/g creatinine	23.9 (19.9–28.8)	1.25 (0.816–1.79)	6.25 (3.73–10.0)	34.9 (26.9–46.6)	114 (101–132)	235 (193–263)	306 (277–342)	1,278
Male								
μg/L	2.96 (2.33–3.77)	< LOD	0.600 (0.400–0.800)	2.30 (1.60–3.10)	13.8 (9.00–18.0)	51.7 (39.7–70.9)	125 (77.2–185)	1,270
μg/g creatinine	2.38 (1.92–2.95)	< LOD	0.513 (0.422–0.654)	1.84 (1.42–2.47)	9.52 (6.79–12.8)	40.4 (31.0–51.5)	90.8 (58.6–125)	1,270

Race/ethnicity^a^								
Mexican American								
μg/L	10.6 (8.08–13.8)	0.500 (0.300–0.800)	1.8 (1.10–2.90)	12.1 (7.00–17.5)	65.9 (43.5–93.3)	247 (174–293)	389 (293–453)	637
μg/g creatinine	9.49 (7.29–12.4)	0.449 (0.268–0.690)	1.43 (0.893–2.23)	11.0 (6.69–16.4)	55.2 (44.9–79.6)	186 (147–271)	329 (194–441)	637
Non-Hispanic black								
μg/L	26.8 (21.5–33.3)	1.60 (1.30–2.10)	6.60 (4.50–8.60)	34.7 (28.3–44.2)	125 (89.5–165)	331 (236–411)	531 (367–730)	678
μg/g creatinine	18.8 (15.2–23.3)	1.14 (0.822–1.41)	4.75 (3.67–6.57)	23.6 (19.0–30.8)	83.7 (61.8–105)	217 (169–255)	318 (242–377)	678
Non-Hispanic white								
μg/L	6.21 (5.02–7.68)	0.300 (< LOD–0.400)	1.00 (0.700–1.30)	6.00 (4.60–9.10)	35.7 (28.4–49.6)	130 (92.1–157)	229 (156–318)	1,038
μg/g creatinine	6.53 (5.32–8.02)	0.286 (< LOD–0.377)	1.01 (0.700–1.36)	6.45 (3.98–9.11)	47.4 (32.6–65.3)	146 (119–184)	241 (184–278)	1,038

CI, confidence interval.

a Participants not defined by the three racial/ethnic groups shown were included only in the total population estimate. LOD = 0.2 μg/L.

**Table 3:** Geometric mean and selected percentiles (95% CIs) of BP concentrations in urine for the U.S. population ≥ 6 years of age: data from NHANES 2005–2006.

Variable	Geometric mean	Percentile						Sample size
		10th	25th	50th	75th	90th	95th	
All								
μg/L	—	< LOD	< LOD	< LOD	1.30 (0.800–1.80)	6.60 (4.80–10.7)	19.6 (16.4–26.7)	2,548
μg/g creatinine	—	< LOD	< LOD	< LOD	1.16 (0.909–1.62)	7.82 (6.26–11.2)	21.2 (16.8–27.3)	2,548

Age group (years)								
6–11								
μg/L	—	< LOD	< LOD	< LOD	0.400 (0.200–0.600)	1.90 (0.800–5.80)	7.50 (1.70–18.5)	356
μg/g creatinine	—	< LOD	< LOD	< LOD	0.442 (0.368–0.663)	1.65 (1.00–5.29)	7.92 (1.64–30.5)	356
12–19								
μg/L	—	< LOD	< LOD	0.200 (< LOD–0.400)	0.0900 (0.600–1.80)	9.60 (4.10–16.1)	24.6 (14.5–33.4)	702
μg/g creatinine	—	< LOD	< LOD	0.194 (< LOD–0.255)	0.0694 (0.486–1.20)	6.67 (3.60–11.2)	18.7 (9.52–28.9)	702
20–59								
μg/L	—	< LOD	< LOD	< LOD	1.80 (0.900–2.50)	6.50 (4.30–11.7)	17.8 (10.1–28.2)	1,040
μg/g creatinine	—	< LOD	< LOD	< LOD	1.48 (1.01–1.97)	7.81 (5.36–13.6)	19.8 (13.0–26.9)	1,040
≥60								
μg/L	—	< LOD	< LOD	< LOD	1.10 (0.400–2.20)	11.8 (5.00–23.5)	41.6 (16.5–92.0)	450
μg/g creatinine	—	< LOD	< LOD	< LOD	1.49 (0.824–2.59)	18.2 (6.05–39.2)	52.8 (20.9–74.4)	450

Sex								
Female								
μg/L	0.904 (0.760–1.07)	< LOD	< LOD	0.500 (0.400–0.700)	3.70 (2.90–4.80)	17.4 (11.7–20.8)	34.9 (28.2–41.1)	1,278
μg/g creatinine	1.06 (0.914–1.24)	< LOD	< LOD	0.750 (0.598–0.857)	4.44 (3.01–6.26)	19.8 (13.5–25.7)	34.7 (26.9–42.7)	1,278
Male								
μg/L	—	< LOD	< LOD	< LOD	0.300 (< LOD–0.300)	1.10 (0.600–2.00)	3.20 (1.90–5.50)	1,270
μg/g creatinine	—	< LOD	< LOD	< LOD	0.292 (< LOD–0.349)	0.0833 (0.565–1.16)	2.28 (1.43–2.91)	1,270

Race/ethnicity^a^								
Mexican American								
μg/L	—	< LOD	< LOD	0.200 (< LOD–0.300)	1.10 (0.700–2.30)	11.3 (6.40–15.6)	27.3 (16.5–35.9)	637
μg/g creatinine	—	< LOD	< LOD	0.240 (< LOD–0.294)	1.21 (0.761–1.68)	9.16 (5.36–15.2)	20.3 (15.8–29.2)	637
Non-Hispanic black								
μg/L	—	< LOD	< LOD	0.300 (0.200–0.400)	2.10 (1.00–3.50)	9.10 (4.40–25.0)	31.8 (10.1–82.6)	678
μg/g creatinine	—	< LOD	< LOD	0.217 (< LOD–0.272)	1.46 (0.746–2.64)	8.18 (3.65–17.9)	21.0 (10.8–51.3)	678
Non-Hispanic white								
μg/L	—	< LOD	< LOD	< LOD	1.00 (0.700–1.80)	5.80 (4.00–9.00)	17.7 (11.7–25.9)	1,038
μg/g creatinine	—	< LOD	< LOD	< LOD	1.12 (0.847–1.65)	6.93 (5.21–9.91)	20.9 (15.4–27.3)	1,038

CI, confidence interval. —, not calculated: proportion of results below the LOD was too high to provide a valid result.

a Participants not defined by the three racial/ethnic groups shown were included only in the total population estimate. LOD = 0.2 μg/L.

**Table 4:** Selected percentiles (95% CIs) of EP concentrations in urine for the U.S. population ≥ 6 years of age: data from NHANES 2005–2006.

Variable	Percentile						Sample size
	10th	25th	50th	75th	90th	95th	
All							
μg/L	< LOD	< LOD	< LOD	4.80 (3.40–6.00)	26.2 (19.4–31.9)	57.2 (40.3–83.4)	2,548
μg/g creatinine	< LOD	< LOD	< LOD	4.61 (3.39–6.30)	27.1 (19.1–34.0)	66.5 (46.5–84.2)	2,548

Age group (years)							
6–11							
μg/L	< LOD	< LOD	< LOD	< LOD	2.60 (1.70–4.70)	9.90 (2.80–30.7)	356
μg/g creatinine	< LOD	< LOD	< LOD	< LOD	4.18 (1.91–10.2)	13.4 (4.18–29.0)	356
12–19							
μg/L	< LOD	< LOD	< LOD	2.30 (1.30–3.20)	11.3 (7.90–24.1)	38.0 (17.7–122)	702
μg/g creatinine	< LOD	< LOD	< LOD	1.94 (1.29–2.65)	10.9 (3.57–20.1)	32.0 (14.1–57.8)	702
20–59							
μg/L	< LOD	< LOD	< LOD	6.00 (4.80–8.10)	29.1 (20.7–37.4)	62.8 (42.4–86.8)	1,040
μg/g creatinine	< LOD	< LOD	< LOD	5.83 (4.63–7.58)	27.5 (18.2–38.2)	70.0 (46.0–91.2)	1,040
≥ 60							
μg/L	< LOD	< LOD	< LOD	5.90 (3.00–12.0)	32.0 (18.9–54.7)	76.4 (32.9–136)	450
μg/g creatinine	< LOD	< LOD	< LOD	7.32 (4.56–11.6)	57.0 (27.5–78.9)	83.4 (50.0–135)	450

Sex							
Female							
μg/L	< LOD	< LOD	1.30 (< LOD–2.20)	10.0 (7.50–13.2)	42.7 (32.3–62.8)	98.7 (74.7–147)	1,278
μg/g creatinine	< LOD	< LOD	2.09 (< LOD–2.60)	12.7 (8.45–16.2)	57.3 (40.7–75.2)	107 (82.9–141)	1,278
Male							
μg/L	< LOD	< LOD	< LOD	1.90 (1.10–3.00)	8.90 (5.80–13.6)	25.2 (16.0–29.2)	1,270
μg/g creatinine	< LOD	< LOD	< LOD	1.82 (1.48–2.15)	6.38 (3.55–9.95)	15.5 (10.2–22.3)	1,270

Race/ethnicity^a^							
Mexican American							
μg/L	< LOD	< LOD	< LOD	3.70 (1.90–6.10)	24.0 (11.6–37.7)	55.6 (40.3–120)	637
μg/g creatinine	< LOD	< LOD	< LOD	3.49 (2.09–5.23)	23.5 (11.8–33.2)	68.5 (32.1–107)	637
Non-Hispanic black							
μg/L	< LOD	< LOD	1.00 (< LOD–1.50)	5.70 (3.70–9.80)	31.3 (20.4–52.1)	92.3 (41.5–166)	678
μg/g creatinine	< LOD	< LOD	0.972 (< LOD–1.14)	4.48 (2.90–6.07)	22.0 (14.1–36.8)	47.5 (33.7–106)	678
Non-Hispanic white							
μg/L	< LOD	< LOD	< LOD	4.80 (3.10–6.10)	24.0 (15.5–32.3)	54.7 (36.4–86.8)	1,038
μg/g creatinine	< LOD	< LOD	< LOD	4.73 (3.38–6.58)	27.5 (16.2–38.4)	70.6 (46.0–91.2)	1,038

CI, confidence interval. Geometric means for EP were not calculated: the proportion of results below the LOD was too high to provide a valid result.

a Participants not defined by the three racial/ethnic groups shown were included only in the total population estimate. LOD = 1.0 μg/L.

**Table 5:** Adjusted LSGM concentrations (95% CIs) of MP and PP in various demographic groups.

	LSGM [μg/L (95% CI)]	
	MP	PP
Household income		
< $20,000	48.6 (40.8–57.9)	6.2 (5.3–7.4)
$20,000–$45,000	49.3 (41.4–58.7)	7.0 (5.6–8.7)
> $45,000	62.1 (52.7–73.1)	9.1 (7.7–10.8)
Sex × race/ethnicity		
Male		
Mexican American		4.3 (3.6–5.3)
Non-Hispanic white		1.9 (1.5–2.3)
Non-Hispanic black		8.9 (6.5–12.2)
Female		
Mexican American		33.6 (27.2–41.6)
Non-Hispanic white		21.1 (16–27.8)
Non-Hispanic black		45.2 (35.8–57.1)
Sex × age		
Male		
6–11 years	26 (15.9–42.3)	2.6 (1.5–4.4)
12–19 years	20.8 (17.2–25.2)	2.2 (1.6–3.0)
20–59 years	24 (19.5–29.5)	2.5 (1.9–3.3)
≥ 60 years	30 (21–42.9)	2.7 (1.6–4.5)
Female		
6–11 years	46.5 (30.7–70.3)	4.5 (2.8–7.2)
12–19 years	74.1 (60.6–90.7)	18.2 (13.2–25.2)
20–59 years	144.6 (113.6–184.1)	31.6 (24.3–41.1)
≥ 60 years	160.2 (129.6–198.1)	28.8 (22.3–37.3)
Race/ethnicity × age		
Mexican American		
6–11 years	39 (27.6–54.9)	3.9 (2.8–5.3)
12–19 years	71 (59.2–85.2)	8.6 (6.6–11.4)
20–59 years	102.7 (84.5–124.7)	16.9 (13.4–21.3)
≥ 60 years	90.4 (59.4–137.5)	8.7 (5.0–15)
Non-Hispanic white		
6–11 years	26.9 (19.4–37.3)	2.4 (1.6–3.6)
12–19 years	28.7 (24.1–34.2)	4.8 (3.4–6.8)
20–59 years	47.7 (38.8–58.6)	7 (5.4–9.1)
≥ 60 years	63.5 (49.3–81.9)	8.3 (6.0–11.6)
Non-Hispanic black		
6–11 years	138.7 (92.7–207.7)	20.3 (14.2–29)
12–19 years	154.5 (116.3–205.2)	23.8 (16.3–34.6)
20–59 years	133.6 (96.7–184.6)	22.3 (16.4–30.5)
≥ 60 years	95.7 (64.3–142.4)	12.5 (7.9–19.8)
